# Identification of Drought-Resistant Response in Proso Millet (*Panicum miliaceum* L.) Root through Physiological and Transcriptomic Analysis

**DOI:** 10.3390/plants13121693

**Published:** 2024-06-19

**Authors:** Panpan Zhang, Binglei Wang, Yaning Guo, Tao Wang, Qian Wei, Yan Luo, Hao Li, Huiping Wu, Xiaolin Wang, Xiong Zhang

**Affiliations:** 1 College of Life Science, Yulin University, Yulin 719000, China; wangbinglei@nwafu.edu.cn (B.W.); guoyaning01308060@163.com (Y.G.); wangtao821223@163.com (T.W.); weiqian1251@163.com (Q.W.); 1994lyyl@nwafu.edu.cn (Y.L.); 13209212642@163.com (H.L.); whp7979@163.com (H.W.); wangxiaolin8304@163.com (X.W.); 2 Dryland Agricultural Engineering Technology Research Center in Northern of Shaanxi, Yulin 719000, China

**Keywords:** proso millet, transcriptome, molecular mechanism, physiological indexes, root

## Abstract

Proso millet (*Panicum miliaceum* L.) is resilient to abiotic stress, especially to drought. However, the mechanisms by which its roots adapt and tolerate salt stress are obscure. In this study, to clarify the molecular mechanism of proso millet in response to drought stress, the physiological indexes and transcriptome in the root of seedlings of the proso millet cultivar ‘Yumi 2’ were analyzed at 0, 0.5, 1.0, 1.5, and 3.0 h of stimulated drought stress by using 20% PEG-6000 and after 24 h of rehydration. The results showed that the SOD activity, POD activity, soluble protein content, MDA, and O_2_^−^· content of ‘Yumi 2’ increased with the time of drought stress, but rapidly decreased after rehydration. Here, 130.46 Gb of clean data from 18 samples were obtained, and the Q30 value of each sample exceeded 92%. Compared with 0 h, the number of differentially expressed genes (DEGs) reached the maximum of 16,105 after 3 h of drought, including 9153 upregulated DEGs and 6952 downregulated DEGs. Gene Ontology and Kyoto Encyclopedia of Genes and Genomes pathway analyses revealed that upregulated DEGs were mainly involved in ATP binding, nucleus, protein serine/threonine phosphatase activity, MAPK signaling pathway–plant, plant–pathogen interactions, and plant hormone signal transduction under drought stress, while downregulated DEGs were mainly involved in metal ion binding, transmembrane transporter activity, and phenylpropanoid biosynthesis. Additionally, 1441 TFs screened from DEGs were clustered into 64 TF families, such as AP2/ERF-ERF, bHLH, WRKY, NAC, MYB, and bZIP TF families. Genes related to physiological traits were closely related to starch and sucrose metabolism, phenylpropanoid biosynthesis, glutathione metabolism, and plant hormone signal transduction. In conclusion, the active oxygen metabolism system and the soluble protein of proso millet root could be regulated by the activity of protein serine/threonine phosphatase. AP2/ERF-ERF, bHLH, WRKY, NAC, MYB, and bZIP TF families were found to be closely associated with drought tolerance in proso millet root. This study will provide data to support a subsequent study on the function of the drought tolerance gene in proso millet.

## 1. Introduction

A significant portion of the global land area, roughly one-third, lies in arid and semi-arid conditions, with nearly half of this ratio found in China. This water scarcity severely impedes crop growth and yield [1], particularly in the early stages of development, where even brief drought spells can lead to substantial reductions in production [2]. Developing drought-resistant varieties is deemed the most effective solution to this challenge. Despite decades of breeding efforts, many crop species have depleted their genetic reservoirs for drought resistance, underscoring the importance of exploring new plant genetic resources with efficient water utilization traits [3,4].

Proso millet (*Panicum miliaceum* L.) emerges as a crucial, high-quality coarse grain resource in China, and is widely cultivated in Asia, Europe, and other continents [5]. Proso millet is often cited as a pioneer plant due to boasting a short growth cycle, low water requirements, and robust abiotic stress resistance [6,7]. Beyond its nutritional richness [8], which contributes significantly to the development of modern functional foods [9], proso millet serves as a vital genetic asset for enhancing crop drought resistance. Nonetheless, the physiological and molecular mechanisms of drought tolerance in proso millet are unclear [10]. Therefore, it is very necessary to deeply study the molecular mechanisms of drought resistance of proso millet.

In recent years, rapid advancements in high-throughput transcriptome sequencing technology have provided insights into the physiological and biochemical molecular changes that occur in plants under drought stress [11,12]. While extensive research has delved into regulating gene composition, expression, and signal transduction in conventional crops, the complexity of drought tolerance mechanisms, inherited through multiple quantitative traits, persists. Considering its exceptional drought resilience, proso millet stands out as a key genetic resource for bolstering the drought resistance of other crops.

Although many researchers pay attention to the molecular mechanism of drought resistance of proso millet [10,13,14,15], most of them focus on the leaves or the entire above-ground part of proso millet. The root is a very important organ of proso millet, which has important functions, such as plant fixation, water and nutrient absorption [16], and nutrient synthesis and storage. When crops are subjected to drought stress, the root is the most sensitive organ [17], which first senses and quickly sends out signals to make the whole plant respond to the stress. At the same time, the root morphological structure and internal physiology have corresponding changes, which affect the above-ground construction and yield [18]. Therefore, this study focuses on proso millet seedling roots, investigating key physiological indices under normal watering conditions and at various intervals following drought stress.

In this study, RNA-seq technology was used to identify differentially expressed genes in response to drought stress, followed by qPT-PCR analysis of the expression of key drought-resistant genes in the root system, by analyzing the transcriptome data of the root system of millet seedlings during different drought treatments. The study aimed to provide a theoretical basis for understanding the molecular mechanism of drought resistance in the proso millet root system and provide valuable genetic resources for improving the drought resistance of major crops by using bioinformatics analysis to elucidate the drought resistance signaling pathways and key drought resistance genes in the root system.

## 2. Results

### 2.1. Physiological Differences in Response to Drought Stress and Re-Watering in Proso Millet

**Figure 1:**
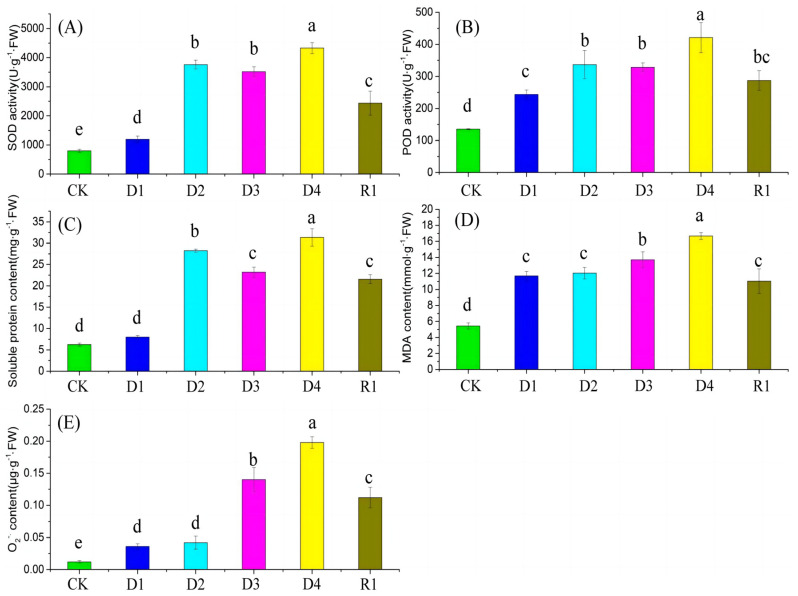
Physiological responses to drought exposure duration. (**A**) SOD: superoxide dismutase, (**B**) POD: peroxidase, (**C**) soluble protein, (**D**) MDA: malondialdehyde, and (**E**) O_2_^−^·: superoxide anion radical. The different lowercase letters indicate significant differences between treatments (Duncan’s test; *p* < 0.05).

To investigate the physiological response of proso millet roots under drought and re-watering conditions, the SOD activity, POD activity, soluble protein content, MDA content, and O_2_^−^· content were examined across five stages (Figure 1 and Table S1). Compared with the control, the SOD activity, POD activity, and soluble protein contents were elevated as drought stress progressed. Outside of soluble proteins at the D1 stage, the SOD activity, POD activity, and soluble protein content were significantly different between D1, D2, D3, and D4 stages and the control. After rehydration, the SOD activity, POD activity, and soluble protein content dramatically dropped. However, the three physiological indices were significantly higher than those at the control stage, which increased by 205.8%, 112.8%, and 246.2%, respectively (Figure 1A–C). MDA and O_2_^−^· contents were significantly different between the control and D1 through D4 and R1, and with an increased drought duration, MDA and O_2_^−^· contents showed an increasing trend. There were no significant differences in MDA content and O_2_^−^· content between D1 and D2, which peaked at the D4, suggesting that MDA and O_2_^−^· were induced by drought stress. At the conclusion of the experiment, the MDA and O_2_^−^· contents were 11.05 mmol·g^−1^·FW and 0.112 μg·g^−1^·FW, respectively, significantly higher than those under the control (Figure 1D,E).

### 2.2. Transcriptome Reprogramming in Proso Millet Root Triggered by Drought Stress and Re-Watering

To explore the molecular mechanism of drought stress induced in proso millet root, 18 samples (encompassing 3 independent biological replicates) for RNA-seq were obtained from the control (0 h), drought stress (0.5 h, 1.0 h, 1.5 h, and 3.0 h), and rehydration, referred to as CK, D1, D2, D3, D4, and R1, correspondingly. Over 0.87 billion high-quality reads were acquired. Following sequencing quality control, a total of 130.46 Gb of clean data was obtained, and the Q30 value of each sample was no less than 92.34%, indicating that the sequencing data were reliable. The GC content was between 53.32% and 55.66% (Table 1). Between 79.11% and 94.07% of reads were mapped to the *Panicum miliaceum* L. reference genome using HISAT2 [19]. An average of 79.22%, 11.35%, 63.69%, and 63.77% of reads were mapped to the unique location, multiple locations, plus chain, and negative chain of the reference genome, respectively. A total of 68,316 transcripts were obtained from all samples, including 55,965 documented and 12,351 novel transcripts.

### 2.3. Functional and Pathway Annotation of Genes

**Figure 2:**
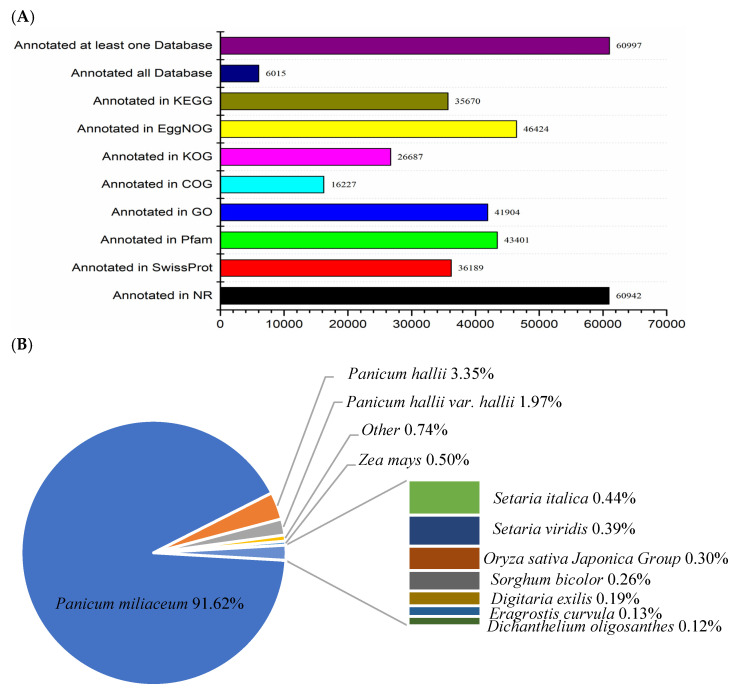
Annotation of proso millet transcript identities and characteristics. (**A**) Unigenes annotated in 8 databases (KEGG, EggNOG, KOG, COG, GO, Pfam, SwissProt, and NR). (**B**) Unigenes distributed into different species.

To acquire genetic function, a total of 68,315 unigenes were annotated into the NR, SwissProt, Pfam, GO, COG, KOG, EggNOG, and KEGG databases. A total of 60,997 unigenes were annotated in at least one database, accounting for 89.2%. There were 6015 unigenes simultaneously annotated across eight databases. The numbers of unigenes in the NR, SwissProt, Pfam, GO, COG, KOG, EggNOG, and KEGG databases were 60,942, 36,108,189, 43,401, 41,904, 16,227, 26,687, 46,424, and 35,670, respectively (Figure 2A and Table S2). In our homologous sequence alignment, there were 55,838 sequences from *Panicum miliaceum* matched, followed by *Panicum hallii*, *Panicum hallii var. hallii*, *Zea mays*, *Setaria italica*, *Setaria viridis*, *Oryza sativa Japonica Group*, *Sorghum bicolor*, *Digitaria exilis*, *Eragrostis curvula*, and *Dichanthelium oligosanthes*. A total of 450 (0.74%) unigenes matched sequences from other species (Figure 2B and Table S2).

### 2.4. DEGs under Drought and Re-Watering Conditions

**Figure 3:**
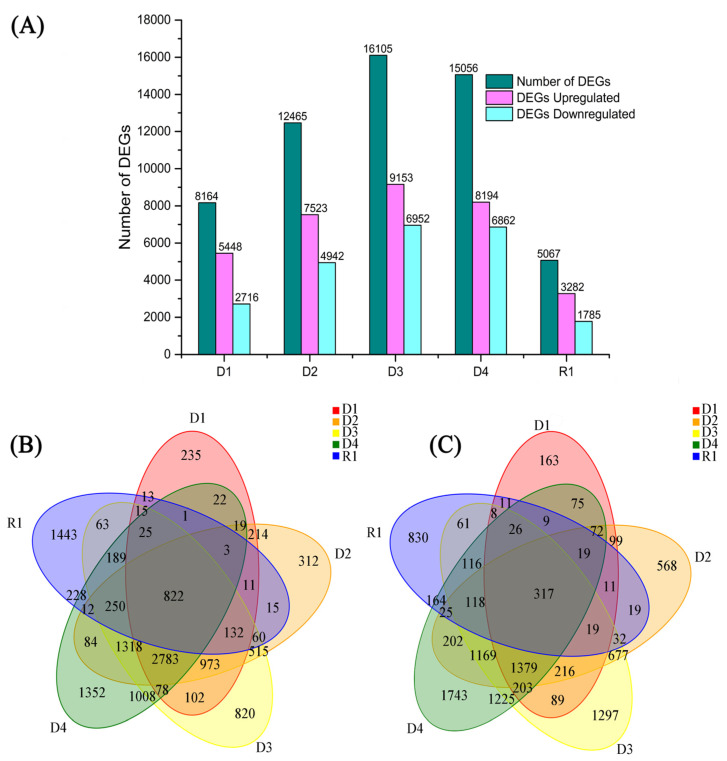
Up- and down-regulated differentially expressed genes (DEGs) and Venn diagrams showing the numbers of DEGs across five comparisons. (**A**) DEGs were upregulated or downregulated by drought–rehydration treatment in proso millet. D1, D2, D3, D4, and R1 show the comparisons of differential unigenes identified between 0.5 h of drought vs. control, 1.0 h of drought vs. control, 1.5 h of drought vs. control, 3.0 h of drought vs. control, and 24 h of rehydration vs. control, respectively. (**B**) Upregulated DEGs in Venn diagrams and (**C**) downregulated DEGs in Venn diagrams.

DEGs were determined using DESeq2, and a fold change ≥ 1.50 and adjusted *p*-value < 0.01 were employed to define significantly differentially expressed genes [16]. We identified 8164 DEGs (5448 upregulated and 2716 downregulated) in CK vs. D1, 12,465 DEGs (7523 upregulated and 4942 downregulated) in CK vs. D2, 16,105 DEGs (9153 upregulated and 6952 downregulated) in CK vs. D3, 15,056 DEGs (8194 upregulated and 6862 downregulated) in CK vs. D4, and 5067 DEGs (3282 upregulated and 1785 downregulated) in CK vs. R1 (Figure 3 and Table S3). The upregulated and downregulated DEGs in 1.5 h of drought were the highest, suggesting different expression patterns of DEGs under varying drought stages.

To identify common individual genes at different physiological stages, the overlap in each comparison was presented using Venn diagrams (Figure 3B,C). The overlap of upregulated and downregulated genes was the highest between 3.0 h of drought and 0 h of drought, indicating plant damage following severe drought. A total of 822 DEGs were upregulated, and 317 DEGs were downregulated, overlapping with 0.5, 1.0, 1.5, and 3.0 h of drought and 24 h of re-watering versus 0 h of drought, respectively, suggesting a shared set of genes was involved in responses to water shortage and re-watering treatment conditions.

### 2.5. GO Enrichment Analysis of DEGs

**Figure 4:**
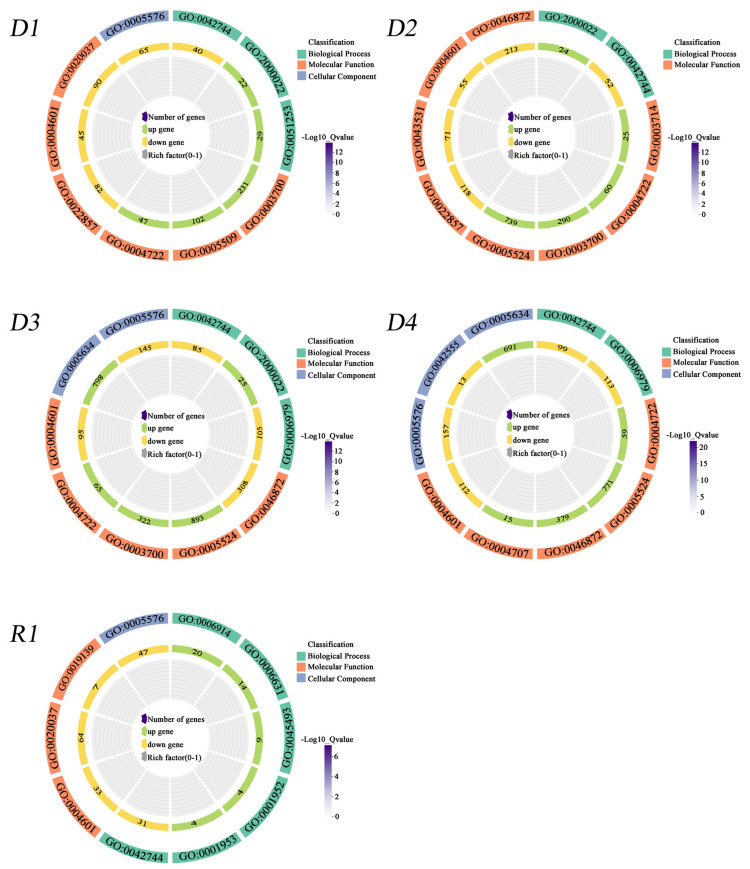
Gene Ontology (GO) enrichment analysis of the most significant up- and down-regulated DEGs (FDR < 0.05). Note: D1~D4, R1: The different groups are CK (0 h), 0.5 h, 1.0 h, 1.5 h, and 3.0 h of drought stress, and 24 h rehydration treatment.

To characterize the primary biological processes involved in drought–rehydration reactions, we analyzed GO enrichment for upregulated and downregulated DEGs across five physiological stages, with FDR < 0.05 (Figure 4 and Table S4). According to the corrected *p*-value, the first five of the most significant accessions to explain the physiological changes are shown in Figure 4. Transcription factor activity, sequence-specific DNA binding, regulation of the jasmonic-acid-mediated signaling pathway, and protein serine/threonine phosphatase activity were enriched in the upregulated DEGs at D1, D2, and D3 stages, while protein serine/threonine phosphatase activity was further enriched at D4, indicating protein phosphorylation in response to drought. Calcium ion binding was enhanced in D1, while ATP binding was enriched in the upregulated DEGs at the D2, D3, and D4 stages, demonstrating that with extended drought stress, DEGs associated with calcium ions weakened and DEGs linked to ATP were activated. The hydrogen peroxide catabolic process, peroxidase activity, and extracellular region were abundant in the downregulated DEGs from D1 to D4 and R1, whereas the DEGs at R1 were the lowest. Autophagy, fatty acid metabolic process, xylan catabolism, regulation of cell-matrix adhesion, and negative regulation of cell-matrix adhesion were abundant in the upregulated DEGs. However, cytokinin dehydrogenase activity was enriched in the downregulated DEGs at R1, suggesting improved fatty and sugar metabolism and decreased cytokinin degradation following re-watering (Figure 4 and Table S4).

### 2.6. Metabolic Pathway Analysis via KEGG

**Figure 5:**
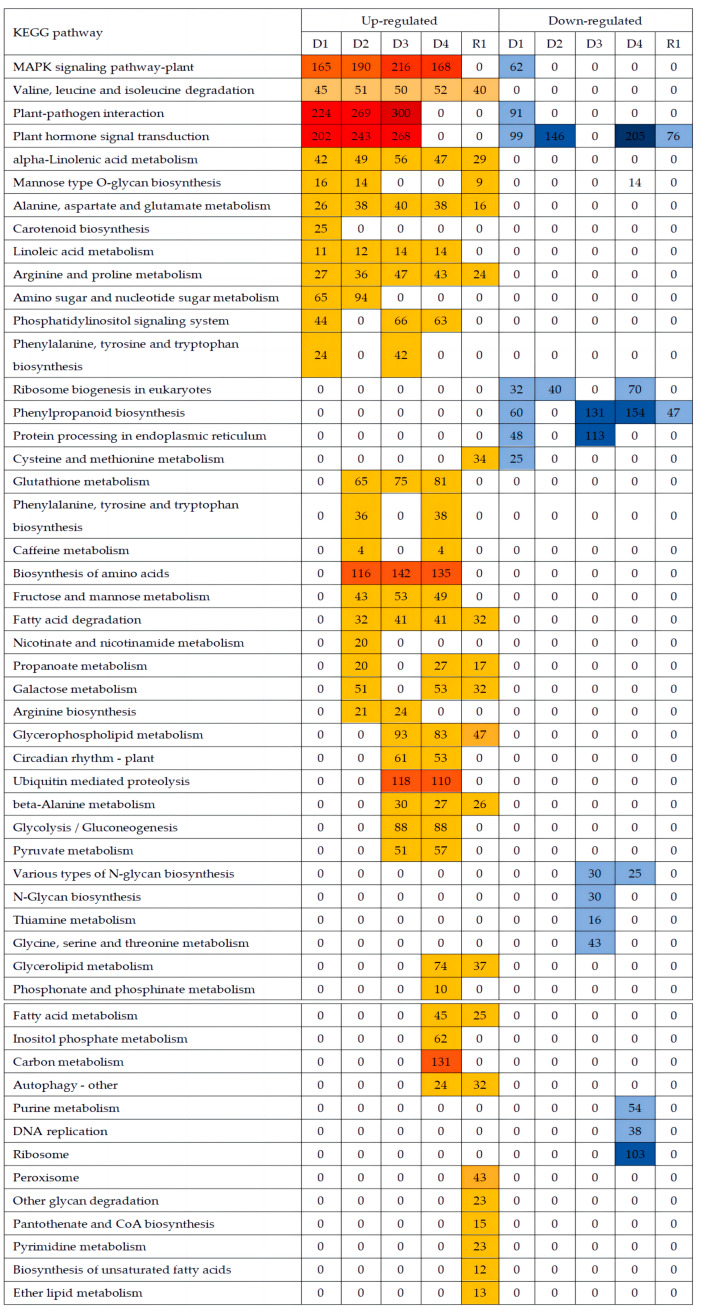
Proso millet metabolic pathways and differentially expressed genes in KEGG analysis under different gradients of drought stress, with FDR < 0.05. Note: D1~D4, R1: The different groups are CK (0 h), 0.5 h, 1.0 h, 1.5 h, and 3.0 h of drought stress, and 24 h rehydration treatment.

The KEGG metabolic pathway analysis results are depicted in Figure 5 and Table S5. Comparing D1 to the CK, 2757 upregulated and 1110 downregulated DEGs were annotated across 128 and 114 different pathways. As drought stress continued, the number of upregulated and downregulated DEGs increased and then decreased. A total of 3963, 4737, and 4292 upregulated genes and 1872, 3151, and 3137 downregulated genes were annotated in the KEGG pathways, involving 133, 135, and 133 different unregulated pathways and 125, 131, and 129 different downregulated pathways. The number of DEGs between the control and following 24 h of rehydration decreased, and 1874 upregulated and 792 downregulated DEGs were involved in 125 and 108 different KEGG pathways (Figure 5 and Table S5). The significantly enriched KEGG pathways are illustrated in Figure 5. The pathways associated with the MAPK signaling pathway, plant–pathogen interaction, and plant hormone signal transduction were enriched in upregulated DEGs between D1 and D3 and downregulated at D1. Valine, leucine, and isoleucine degradation, alpha-linolenic acid metabolism, alanine, aspartate, and glutamate metabolism, linoleic acid metabolism, arginine, and proline metabolism were enriched in upregulated DEGs at D1 to D4, and R1, while ribosome biogenesis in eukaryotes, phenylpropanoid biosynthesis, and protein processing in endoplasmic reticulum were found in downregulated DEGs at D1. The downregulated DEGs associated with nitrogen metabolism were enriched in N-Glycan biosynthesis, thiamine metabolism, glycine, serine, and threonine metabolism at D3, and purine metabolism, DNA replication, and ribosome functions at D4. The upregulated DEGs associated with metabolism were enriched in circadian rhythm–plant, ubiquitin-mediated proteolysis, glycolysis/gluconeogenesis, and pyruvate metabolism at D3 and D4, and glycerolipid metabolism, fatty acid metabolism, and autophagy–other at D4 and R1, indicating improved metabolism in response to drought and re-watering. The pathways associated with cysteine and methionine metabolism peroxisome, other glycan degradation, pantothenate and CoA biosynthesis, pyrimidine metabolism, biosynthesis of unsaturated fatty acids, and ether lipid metabolism were only enriched at R1, indicating that biosynthesis became active following re-watering.

### 2.7. Drought Stress and Rehydration-Responsive Transcriptional Factors

**Figure 6:**
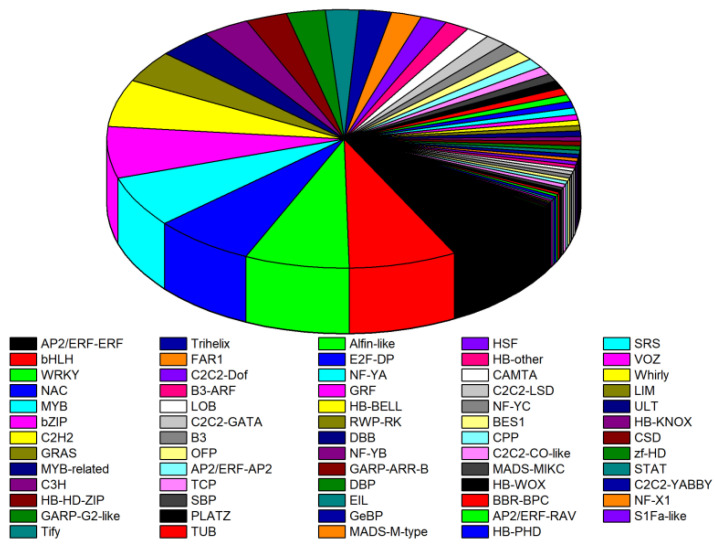
Analysis of TFs in DEGs. Different colors indicate various TF families identified from DEGs across five stages.

A total of 1441 transcription factors spanning 64 families of TFs were included in the DEGs during drought stress and re-watering, as visualized in Figure 6 and Table S6. The six major TF families were AP2/ERF-ERF, bHLH, WRKY, NAC, MYB, and bZIP, accounting for 8.12%, 7.36%, 7.15%, 6.87%, 6.45%, and 6.45% of the total TFs. There were C2H2, GRAS, MYB-related, and C3H families accounting for above 3% and below 6% of the total TFs, while the remaining 54 families all represented below 3%. Totals of 62, 67, 84, 58, and 13 TFs among 543, 657, 729, 575, and 230 TFs in AP2/ERF-ERF were upregulated at D1, D2, D3, D4, and R1, respectively (Figure 6 and Table S6). The 43 upregulated and 16 downregulated TFs in the bHLH family increased to 49 upregulated and 29 downregulated TFs from D1 to D4, while the numbers of upregulated and downregulated TFs decreased after rehydration. During drought and rehydration, the levels of upregulated TFs tended to increase and then decrease in the WRKY family, with the largest number being reached at D3.

### 2.8. Weighted Gene Co-Expression Network Analysis

**Figure 7:**
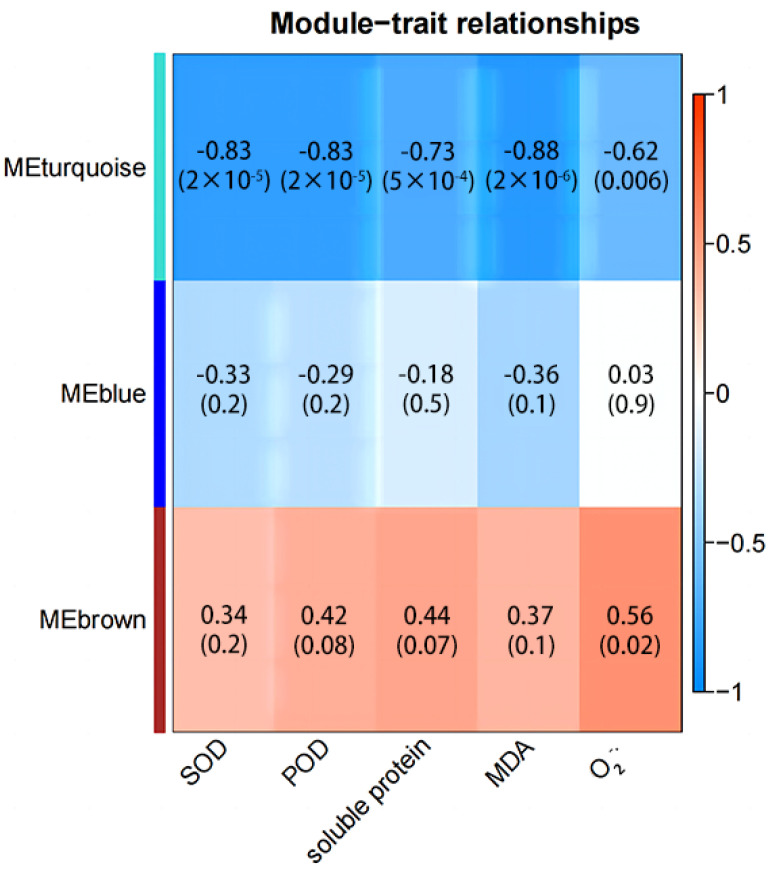
Module–trait relationships with physiological indices. The numbers indicate the correlation coefficients of modules with physiological indexes. The numbers in brackets are the *p*-values.

Weighted gene co-expression network analysis was employed to analyze gene co-expression profiles of proso millet under water restriction and fluid rehydration conditions, and to identify the relationship between genes and physiological indices. Three co-expression modules and their correlation coefficients were characterized and obtained (Figure 7). The five physiological indices were positively correlated with the brown module, and the correlation coefficient of O_2_^−^· content was the highest (0.56). The brown module was primarily enriched in propanoate metabolism, beta-alanine metabolism, and starch and sucrose metabolism (Table S7), suggesting that starch and sucrose metabolism participated in regulating O_2_^−^· content in proso millet. However, SOD, POD, soluble protein, MDA, and O_2_^−^· were negatively correlated with the turquoise module, with a correlation coefficient of 0.62–0.88. The turquoise module was predominantly enriched in phenylpropanoid biosynthesis, glutathione metabolism, linoleic acid metabolism, and plant hormone signal transduction, which inferred that the active oxygen metabolism system and soluble protein were influenced by phenylpropanoid biosynthesis, glutathione metabolism, and plant hormone signal transduction.

### 2.9. Validation of RNA-Seq Data Using qRT-PCR

**Figure 8:**
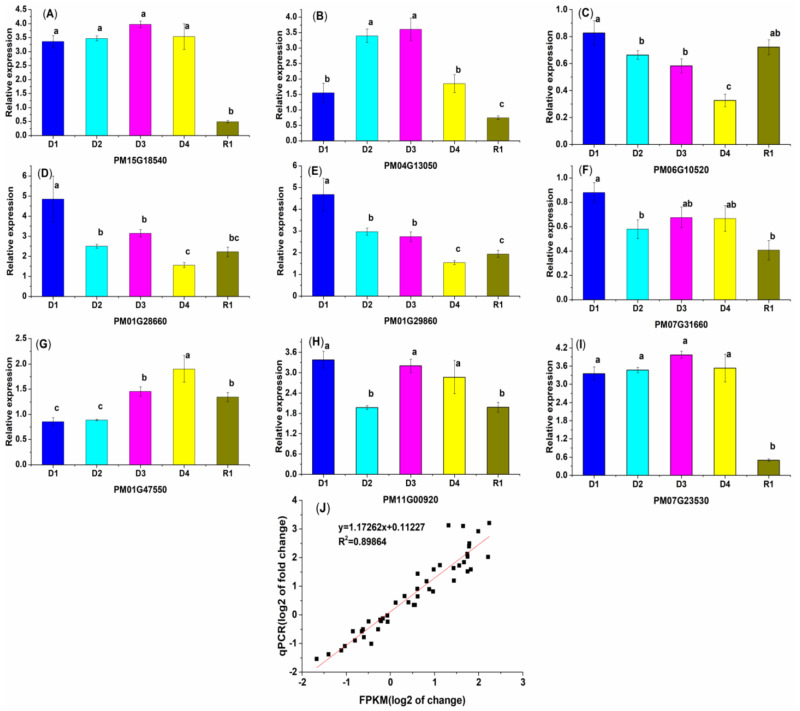
The qRT-PCR analysis of the relative expression levels of selected genes across different stages. (**A**–**I**) Relative expression of 9 genes. D1~D4, R1: The different groups are CK (0 h), 0.5 h, 1.0 h, 1.5 h, and 3.0 h of drought stress, and 24 h rehydration treatment. (**J**) Comparison between the relative expressions obtained from qRT-PCR and RNA-seq. The X-axis is the log^2^ value of relative expression and the Y-axis is the log^2^ value of FPKM in RNA-seq. Data are shown as the mean ± SD (*n* = 3). Different letters in each figure indicate significant differences at the 0.01 level.

To verify the accuracy of the RNA-seq analysis, qRT-PCR analysis was employed to uncover the nine randomly selected gene expressions under different drought stresses, from bHLH, WRKY, NAC, and bZIP. The relative expression trend of the chosen genes detected by qRT-PCR was positively correlated with the RNA-seq data, suggesting that the RNA-seq result was accurate. The relative expression levels of *PM15G18540*, *PM04G13050*, and *PM07G23530* increased gradually at the D1 to D3 stages, decreased slowly at the D4 stage, and decreased rapidly at R1. The levels of *PM06G10520*, *PM01G28660*, and *PM01G29860* expression were highest at D1 and were lowest at D4. In the R1 stage, the relative expression of *PM06G10520* was higher than under D2, and the expression of *PM01G28660* and *PM01G29860* at R1 was higher than at D4. From D1 to R1, compared to CK, *PM07G31660* was downregulated, but *PM11G00920* was upregulated, while *PM01G47550* showed the highest relative expression at D4, and the lowest relative expression at D1 (Figure 8 and Table S8).

## 3. Discussion

The roots are the primary water absorption organ of crops, and their growth and development have a crucial function in maintaining crop viability under drought stress [20]. Drought tolerance of roots is a complex physiological and biochemical process controlled and regulated by various genes, metabolic pathways, and TFs.

### 3.1. Response of Physiological Traits to Drought and Rehydration Conditions

The accumulation of reactive oxygen species, including singlet oxygen, superoxide anion, hydrogen peroxide, and hydroxyl radical, produces membrane lipid peroxidation and seriously damages plant cells when plants encounter drought stress [21]. The antioxidant defense system comprising antioxidant enzymes is an essential mechanism allowing plants to remove excess ROS and protect against oxidative stress [22]. SOD and POD exist widely in plant cells, regulating the ROS concentration by directly removing oxides [23]. In this study, with an extended drought duration, SOD and POD exhibited a trend of rapid increase followed by a slow increase (Figure 1). Following the re-watering stage, SOD and POD exhibited a rapid decline. The accumulation of soluble substances is effective for resisting drought stress and maintaining an osmotic balance [24]. Compared to the control, the soluble protein content in proso millet root increased under drought stress and decreased under rehydration, because abiotic stress inhibits protein synthesis [25]. MDA can reflect the level of lipid peroxidation of plant cell membranes and is often employed to evaluate the tolerance of plants to biological or abiotic stresses [26]. Under drought stress for 3 h, the MDA and O_2_^−^· contents were higher than those for the control and rehydration stages, probably due to the excessive ROS resulting in higher lipid membrane peroxidation [27]. These findings indicate that proso millet roots resist and adapt to drought stress by regulating the root physiology.

### 3.2. DEGs Reflect Drought and Rehydration Conditions

When plants experience stress, they respond at the gene level by mobilizing numerous genes into a complex regulatory network to produce corresponding proteins, so as to control metabolite synthesis and regulate the mechanism balance of plants [28]. In this study, the root transcription levels of proso millet following 0, 0.5, 1.0, 1.5, and 3.0 h of drought stress treatment and 24 h of rehydration were analyzed, and a fold change ≥ 1.50 and *p*-value < 0.01 were employed as screening criteria. Compared to the control, there were 8164 (5448 upregulated and 2716 downregulated), 12,465 (7523 upregulated and 4942 downregulated), 16,105 (9153 upregulated and 6952 downregulated), and 15,056 (8194 upregulated and 6862 downregulated) DEGs after 0.5, 1.0, 1.5, and 3.0 h, respectively (Figure 3A), suggesting that drought stress mainly stimulated the number of upregulated DEGs. This is in contrast with previous studies by Hou et al. [29] in *Fagopyrum esculentum* seedlings and Shao et al. [30] in Bermuda grass, potentially caused by the strong drought resistance of proso millet. Under the re-watering conditions, the number of DEGs decreased significantly, consistent with Zhou et al. [31] and their findings in giant juncao, suggesting that rehydration restored some of the DEGs to control levels.

### 3.3. Metabolic Pathway Response to Drought and Rehydration Conditions

Drought stress is a severe environmental stress that impacts global crop production. Maize adapts to drought stress by modulating the activity of serine/threonine protein phosphatase type-2C (PP2C) [32]. Alfalfa MP2C can regulate the MAPK pathway, participate in the phosphorylation and dephosphorylation process, and act as a regulator of stress signals to cope with stress [33]. In this study, GO enrichment findings indicated that upregulated DEGs were significantly enriched in ATP binding (GO:0005524), transcription factor activity, sequence-specific DNA binding (GO:0003700), and protein serine/threonine phosphatase activity (GO:0004722) between 1 and 3 h after drought (Figure 4 and Table S4). However, the upregulated differentially expressed genes were significantly enriched in calcium ions (GO:0005509) 0.5 h after drought (Figure 4 and Table S4), suggesting that Ca^2+^ plays an important role in the early drought resistance of plants [34]. KEGG enrichment exhibited that the upregulated DEGs were significantly enriched in MAPK signaling pathway–plant, plant–pathogen interactions, plant hormone signal transduction, biosynthesis of amino acids, ubiquitin-mediated proteolysis, and carbon metabolism, and the downregulated DEGs were significantly enriched in phenylpropanoid biosynthesis under drought stress (Figure 5 and Table S5). The number of DEGs enriched in the phenylpropanoid biosynthesis pathway of the roots was the largest, primarily involved in the anabolic pathway of lignin [35]. The downregulated expression of genes associated with the synthesis of phenylpropane compounds significantly slowed down the synthesis of lignin [36], and the synthesis of lignin is an energy-intensive and irreversible process [37]. Therefore, this may be one of the strategies to economize energy and carbon in the short-term response to drought stress in proso millet roots. According to the correlation between modules and traits, phenylpropanoid biosynthesis was negatively correlated with active oxygen metabolism and soluble protein synthesis (Figure 7 and Table S7), confirming phenylpropanoid metabolic involvement in plant development processes and responses to diverse biological and abiotic stresses [38].

### 3.4. TFs Regulate Drought Stress

TFs are DNA-binding proteins that promote or inhibit the expression of target genes by specifically binding to *cis*-elements in promoters. TFs play crucial roles in biological processes, such as growth and development, cell cycle regulation, transcriptional regulation, and response to environmental stresses [39,40]. Many TF families, including MYB, ARC, WRKY, GRAS, and AP2, play key roles in plant responses to abiotic stress [41,42]. In our study, TFs were primarily distributed across AP2/ERF-ERF, bHLH, WRKY, NAC, MYB, and bZIP families. AP2/ERF is a family of plant-specific transcription factors found in various plants, including *Arabidopsis*, rice, and soybeans [43]. This study indicated that the AP2/ERF-ERF family contained 62, 67, 84, and 58 upregulated DEGs after 0.5, 1.0, 1.5, and 3.0 h of drought stress, respectively (Figure 6 and Table S6). Under drought stress, AP2/ERF transcription factor expression levels are low, and hormone or stress-related genes combine with conserved AP2/ERF transcription factor elements and expression regulation [44]. The WRKY gene family is widely distributed in plants and is one of the most abundant and functional transcription factors [45]. Moreover, the *TaWRKY10* gene plays an important role in wheat under drought stress responses [46]. A total of 32 *PmWRKY* genes were identified, and *OsWRKY47* had higher expression levels in the root than in the leaf [47]. The upregulated DEGs were more abundant than the downregulated DEGs in the WRKY and bHLH families after 0.5 to 3.0 h of drought stress. Therefore, we concluded that the upregulated DEGs of WRKY and bHLH transcription factors may play a positive role in the resistance of proso millet root to drought stress.

## 4. Materials and Methods

### 4.1. Plant Material and Drought–Rehydration Treatment

The proso millet variety employed in this experiment was Yumi 2, provided by the Small Grain Laboratory of Northwest A&F University. Healthy and complete glutinous proso millet seeds were chosen for seed treatment. These seeds were disinfected with 10% sodium hypochlorite for 15 min, rinsed three times with distilled water, and soaked in water at 25 °C for 24 h until the radicle broke through the seed coat. Prior to hydroponic treatment, the seeds with accelerated germination were placed in an incubator with two layers of filter paper, during which an appropriate amount of water was sprayed. After three days, seedlings with the same germination status were chosen and placed in a 0.6 m × 0.3 m × 0.1 m culture frame.

Three liters of Hoagland nutrient solution were added to each culture frame, and a perforated foam board was fitted over the nutrient solution. In addition, the foam board was wrapped with tinfoil to create darkness for the roots. A total of 12 holes were evenly distributed on the board, and 1 proso millet seedling was placed within each hole. After transplanting, the culture frame was moved to an artificial climate box, and each culture frame was connected to an air pump, ensuring sufficient dissolved oxygen for seedling roots. The light intensity in the artificial climate box (Ningbo Ledian Instrument Manufacturing Co., Ltd., GLD-1000D-4) was established at 30,000 lux, with a photoperiod of 16 h of light/8 h of darkness and temperature maintained at 25 °C in light/18 °C in darkness. At the three-leaf stage, proso millet seedlings were placed into Hoagland nutrient solution containing 20% (*w*/*v*) PEG-6000 (−0.6 MPa of water potential) supplied by Guangdong Guanghua Sci-Tech Co., Ltd., and the plants were sampled at 0 h (CK), 0.5 h (D1), 1 h (D2), 1.5 h (D3), and 3 h (D4) after treatment, and 24 h after rehydration (R1). Three biological replicates were harvested for physiological tests and RNA-seq analysis. All samples were flash-frozen in liquid nitrogen for half an hour following collection and kept at −80 °C for subsequent use.

### 4.2. Measurements of Root Physiological Index

To assess the physiological indices of proso millet seedlings, three representative plants were chosen from each treatment, and the roots were rapidly rinsed. According to previously reported methods, 0.5 g of fresh root samples was rapidly homogenized with 8 mL of ice-cold PBS (50 mmol·L^−1^, pH 7.8). After centrifugation at 12,000× *g* for 30 min at 2 °C, the supernatant was employed to determine the physiological indices via a colorimetric method, encompassing superoxide dismutase (SOD) activity, peroxidase (POD) activity, soluble protein, malondialdehyde (MDA) content, and superoxide anion free radical (O_2_^−^) content. The chemicals used in the root physiological indexes were supplied by Thermo Fisher Scientific Company.

SOD activity was determined as the amount of enzyme that inhibited the nitroblue tetrazolium (NBT) reduction rate by 50% per unit [48]. One unit (U) of POD activity was defined as a change of 1 at 470 nm per minute and the results were expressed as U/g FW [48]. Soluble protein content was determined using previously described methods [49]. The mixture consisted of 0.1 mL of supernatant, 0.9 mL of distilled water, and 5 mL of coomassie brilliant blue, and the absorbance of the mixture was measured at 595 nm.

MDA content was determined using previously described methods [50], and slightly adjusted. Here, 1.5 mL of supernatant was mixed with 2.5 mL of 0.5% thiobarbituric acid, and the mixture was accurately heated in a boiling water bath at 100 °C for 20 min. After cooling, the supernatant was centrifuged again. The absorbance of the supernatant was measured at 450, 532, and 600 nm. O_2_^−^· content was determined using previously described methods [51]. The mixture consisted of 0.5 mL of supernatant, 0.5 mL of PBS, 1.5 mL of 1 mmol/L hydroxylamine hydrochloride, 2 mL of 17 mmol/L p-aminobenzene sulfonic acid, and 2 mL of 7 mmol/L alpha-naphthylamine, and the absorbance of the mixture was measured at 530 nm.

### 4.3. RNA Extraction and Transcriptome Sequencing

Following the manufacturer’s instructions, total RNA from the collected root tissues was isolated using a plant RNA Kit (TianGen, Beijing, China). The integrity of the extracted RNA was determined via agarose gel electrophoresis using an Agilent Bioanalyzer 2100 (Beijing Longyue Biotechnology Development Co., Ltd., Beijing, China). The quality of the extracted RNA was acceptable for library construction, with values of RIN ≥ 7.5 and 28S/16S ≥ 1.8. According to synthetic sequencing (SBS) technology, the libraries were sequenced using an Illumina Hiseq2500 sequencing platform (Illumina, San Diego, CA, USA). High-quality clean reads were acquired through a rigorous filtration step, including the removal of adapter sequences, reads containing over 10% unknown bases, and low-quality reads. Correspondingly, GC content, Q30, and sequence repetition levels of clean data were determined. The proso millet reference genome was downloaded from NCBI. The raw data were deposited in CNCB-NGDC GSA under the accession number subCRA024924.

### 4.4. Gene Functional Annotation

To characterize the functional annotation, the assembled unigenes were analyzed using eight databases, including the NCBI non-redundant protein sequences database (NR), SwissProt protein database, Cluster of Orthologous Groups of proteins (COG) database, and eukaryotic Cluster of Orthologous Groups of proteins (KOG) database.

### 4.5. Evolutionary Genealogy of Genes

The non-supervised Orthologous Groups (EggNOG) database, Kyoto Encyclopedia of Genes and Genomes (KEGG) database using BLASTX toll with E-value < 10^−5^ [51], Gene Ontology (GO) database with the Blast2GO program-based NR annotation with E-value < 10^−10^ [19], and protein family (Pfam) database with an E-value < 10^−2^ were used.

### 4.6. Differentially Expressed Genes Analysis

The clean reads and reference genomes (*Panicum_miliaceum*. GCA_003046395.2_Pm_0390_v2. genome. fa) were quickly and accurately contrasted using HISAT2 v2.2.0 software [52]. StringTie was used to assemble the above reads [53]. During assembly and clustering, the expression levels of unigenes were normalized and determined as the number of fragments per kilobase of transcript per million fragments mapped (FPKM) [54]. Using the Benjamini–Hochberg method, the *p*-values from DESeq2 package analyses were adjusted to control the false discovery rate (FDR) [55]. In this study, DESeq2 v1.44.0 software with a fold change ≥ 1.50 and adjusted *p*-value < 0.01 between different samples was employed for identifying the DEGs [56].

The GOseq R package was employed to perform GO enrichment analysis on DEGs to determine the biological significance of DEGs [57]. The corrected *p*-value was required to be no less than 0.05. The KEGG database was used to analyze various metabolic pathways accounted for by DEGs. KOBAS v3.0 software was used for statistical enrichment tests on DEGs, and corrected *p*-values of <0.05 were considered significantly enriched KEGG entries [58,59].

### 4.7. Transcription Factor Analysis of DEGs

The PlantTFcat tool was used to identify the plant regulatory elements of transcription factors, transcriptional regulators, and protein kinases [60].

### 4.8. Weighted Gene Co-Expression Network Analysis

The relationship between genes and physiological indices was explored using weighted gene co-expression network analysis (WGCNA) [61]. The parameter was established with an expression threshold greater than 1, a module similarity threshold of 0.25, and at least 30 genes within each module.

### 4.9. Quantitative RT-PCR Analysis

To validate the results from RNA-seq, qRT-PCR was conducted with the same RNA-seq assayed samples used in the three biological and technical replicates. Total RNA was extracted using Trizol, in accordance with the manufacturer’s protocol, and then reverse transcribed into cDNA [27]. RNA integrity was assessed using an Agilent Bioanalyzer 7500 (Agilent Technologies, Santa Clara, CA, USA). The proso millet 18 s gene was employed as an internal reference gene [14]. The qRT-PCR cycling was as follows: 30 s at 95 °C, followed by 40 cycles of 5 s at 94 °C and 30 s at 60 °C. The qRT-PCR primers for the chosen genes were designed using Oligo 7 to ensure specificity through melting peaks and dissociation curves (Table 2). The relative expression levels of the selected genes were determined using the 2^−ΔΔCT^ method [15].

### 4.10. Statistical Analysis

Statistical analysis of physiological indicators was conducted with SPSS v19.0 software (SPSS Inc., Chicago, IL, USA) [62]. One-way ANOVA and Duncan’s multiple comparison analysis (*p* < 0.05) were employed to assess the significance of the differences among different treatments [31]. All data were acquired in triplicate.

## 5. Conclusions

Proso millet possesses a high adaptability to drought stress. In this study, the physiology and transcriptome of proso millet root were examined under varying drought stress and re-watering durations. SOD activity, POD activity, soluble protein, MDA, and O_2_^−^· content increased with the time of drought stress, but rapidly decreased following rehydration. Altogether, 16,105 differentially expressed genes were identified after 3 h of drought stress. Differential gene function enrichment analysis revealed that the upregulated DEGs for ATP binding and protein serine/threonine phosphatase activity and the downregulated DEGs for phenylpropane synthesis are important regulatory pathways for drought stress resistance in proso millet root. Important transcription factors for drought stress resistance in proso millet roots included AP2/ERF-ERF, bHLH, WRKY, NAC, MYB, and bZIP families. Physiological indicators and RNA-seq results provided data support for subsequent drought tolerance gene function studies in proso millet.

## Figures and Tables

**Table 1:** Summary of sequencing reads after filtering and genome mapping.

Sample	Total Clean Reads (Mb)	Total Clean Bases (Gb)	Clean Reads (Q30, %)	GC Ratio (%)	Mapped Reads (Mb)	Uniq Mapped Reads (Mb)	Multiple Mapped Reads (Mb)	Reads Mapped to ‘+’ (Mb)	Reads Mapped to ‘−’ (Mb)
CK-1	44.53	6.65	93.62	54.16	40.88 (91.81%)	35.53 (79.77%)	5.36 (12.03%)	29.24 (65.67%)	29.28 (65.75%)
CK-2	49.64	7.42	93.46	54.07	45.81 (92.28%)	40.10 (80.79%)	5.71(11.50%)	32.26 (64.99%)	32.30 (65.06%)
CK-3	50.10	7.49	93.71	54.50	47.12 (94.07%)	41.29 (82.43%)	5.83 (11.64%)	33.11 (66.10%)	33.14 (66.16%)
D1-1	56.70	8.46	92.86	54.93	51.81 (91.38%)	44.94 (79.26%)	6.87 (12.12%)	37.13 (65.49%)	37.19 (65.59%)
D1-2	44.14	6.58	92.69	55.29	41.15 (93.22%)	35.66 (80.78%)	5.49(12.44%)	29.60 (67.05%)	29.65(67.16%)
D1-3	54.94	8.21	93.22	55.47	51.39 (93.54%)	44.79 (81.53%)	6.60 (12.01%)	36.43 (66.31%)	36.48 (66.40%)
D2-1	42.38	6.33	93.17	55.66	38.83 (91.63%)	33.51 (79.07%)	5.32 (12.55%)	28.10 (66.31%)	28.15 (66.42%)
D2-2	46.59	6.96	93.04	55.40	41.21 (88.46%)	35.97 (77.21%)	5.24 (11.25%)	29.07 (62.40%)	29.11(62.48%)
D2-3	49.17	7.34	93.34	55.60	45.00 (91.53%)	38.84 (78.99%)	6.16 (12.53%)	32.50 (66.10%)	32.54 (66.18%)
D3-1	59.94	8.95	93.20	55.03	55.04 (91.82%)	47.50 (79.24%)	7.54 (12.58%)	39.80(66.40%)	39.87 (66.50%)
D3-2	40.98	6.12	93.37	55.43	37.99 (92.71%)	32.84 (80.13%)	5.16 (12.58%)	27.47 (67.04%)	27.51 (67.15%)
D3-3	48.60	7.26	93.30	55.12	43.63 (89.77%)	37.81 (77.79%)	5.83 (11.99%)	31.24 (64.29%)	31.29 (64.38%)
D4-1	50.28	7.52	93.12	54.92	45.66 (90.82%)	39.72(79.01%)	5.94 (11.81%)	32.51 (64.66%)	32.56 (64.76%)
D4-2	59.95	8.96	92.85	54.55	54.61 (91.08%)	47.55 (79.32%)	7.05 (11.76%)	38.76 (64.65%)	38.82(64.76%)
D4-3	47.46	7.10	93.07	55.32	43.53 (91.73%)	38.05 (80.18%)	5.48 (11.55%)	30.74 (64.77%)	30.79 (64.87%)
R1-1	43.50	6.51	92.34	54.30	38.88 (89.37%)	35.90 (82.51%)	2.98 (6.85%)	23.76 (54.61%)	23.79 (54.68%)
R1-2	40.37	6.04	93.14	54.11	34.72 (86.00%)	30.98 (76.74%)	3.74 (9.26%)	23.28 (57.67%)	23.31 (57.74%)
R1-3	43.96	6.57	92.60	53.32	34.78 (79.11%)	31.34 (71.29%)	3.43 (7.81%)	22.78 (51.82%)	22.81 (51.88%)
Average	48.51	7.25	93.12	54.84					

CK represents 0 h of drought stress, D1, D2, D3, and D4 represent 0.5 h, 1.0 h, 1.5 h, and 3.0 h of drought stress, respectively, R1 represents 24 h of rehydration, and three biological repetitions were included in each sample. Total reads were the clean reads collected from sequencing. Total bases were the clean bases collected from sequencing. Q30 represents the percentage of clean data quality value greater than or equal to 30 bases. GC ratio represents the percentage of G and C bases in the total base in the clean data. Mapped reads represent the reads that were matched to the genome of proso millet. Uniq mapped reads are the reads matched to a unique location in the reference genome, and multiple mapped reads indicates that one read matched to multiple locations in the genome. Reads mapped to ‘+’ mean reads matched to a positive sense of genome, and reads mapped to ‘−’ mean reads matched to a negative sense of genome.

**Table 2:** Primers for qRT-PCR.

Name	Forward Primer (5′→3′)	Reverse Primer (5′→3′)	Product Size (bp)
*PM15G18540*	CAAGTCACCACCACCGCTTCTTC	GCTGCTCCCTATCTCTCCCTATCC	113
*PM04G13050*	AACGCCAACAAGACGGACAAGG	CAGCACCTCCATCAACGGCATC	144
*PM06G10520*	TCATCTCTCCCGACCCTCTTTCTTC	GCTGGTAGTGGTGGTGGTATTGC	131
*PM01G28660*	GAGCCAGAACTACGCCGAT	CATTCATCTCCGTCCACAGC	326
*PM01G29860*	CCTCACCTCACACCCATCTCTCC	CGGCTCGTCCATCACCATGTTG	147
*PM07G31660*	ACGACACCAACTCCATCATGTTCTC	GGGCATCTCCAACGACGACTTG	131
*PM01G47550*	AAAGGGATCGGATCGGGAGAGATG	GTTAGTAGCGGCGGCGTTAGC	80
*PM11G00920*	CTCTCGCCCACCAAGGATTAACG	TCTGAGCAACCAAGAATCTCGTGTG	83
*PM07G23530*	CAGTACACACGCACACCACCTC	CATGGCTGTCTGGTCTGGTTTGG	128
*18S*	CGTCGCGTCCACCCTTTG	GATTTGAAGGTTCCAACTTTG	195

## Data Availability

Data are contained within the article.

## Supplementary Materials

The following supporting information can be downloaded at: https://www.mdpi.com/article/10.3390/plants13121693/s1, Table S1: Raw data for the physiological response of proso millet root. Table S2: Annotation of proso millet transcript identities and characteristics. Table S3: Up- and down-regulated DEGs and Venn diagrams. Table S4: GO enrichment of DEGs. Table S5: DEGs in the KEGG pathway. Table S6: TF classification of DEGs. Table S7: Genes in different modules. Table S8: Raw data for qRT-PCR.
